# Symptoms of depression in autistic children and adolescents

**DOI:** 10.3389/fpsyt.2025.1697147

**Published:** 2025-12-15

**Authors:** Elizabeth Valles-Capetillo, Paula Argueta, Lucas A. Martin, Sarah O’Kelley, Rajesh K. Kana

**Affiliations:** Department of Psychology, University of Alabama at Birmingham, Birmingham, AL, United States

**Keywords:** depression, autism, social communication, social interaction, emotional regulation

## Abstract

Depression is a frequently co-occurring condition in autism spectrum disorder (ASD). Notably, autistic individuals are approximately four times more likely to experience depression than the general population. Difficulties in emotion regulation, inhibitory control, and social skills, common in autistic individuals, are thought to contribute to increased risk and vulnerability to depression. The present study sought to examine the prevalence of depressive symptoms and its cognitive correlates in a sample of autistic and neurotypical (NT) children and adolescents (53 ASD and 43 NT). Data were collected using parent-report questionnaires that assessed depressive symptoms, executive functions, and social skills. Group differences and the relationship between depressive symptoms and cognitive measures were assessed by t-test and linear regression analyses respectively. The results indicated a significant increase in depressive symptoms among the autistic, compared to NT, participants (p < 0.0001). Moreover, emotion regulation and social communication and interaction were significantly associated with depressive symptoms in both groups (p < 0.05). Additional analysis revealed that parental stress did not influence the relationship between executive functioning and interaction and depressive symptoms in both groups. But parental stress influenced the relationship between social communication and interaction and depressive symptoms. The present study highlights the dual contributions of emotional regulation and SCI to depression in both autistic and NT population. Furthermore, underscore the importance of addressing emotion regulation and social communication in identifying risk factors of depression and developing intervention strategies for depression.

## 1 Introduction

Depression commonly co-occurs in autism spectrum disorder (ASD), yet accurately screening and diagnosing depression can be challenging due to overlapping symptoms and limited knowledge about the course and its co-occurrence in autistic youth (1–4). Depression is characterized by a range of symptoms, including feelings of sadness, unhappiness, changes in appetite and weight, trouble sleeping, feelings of worthlessness, difficulties with concentration and/or making decisions, and thoughts of death or suicide (5). In the United States, approximately 18.4% of adults report a diagnosis of depression (6). Among children ages 3–17 years, about 4% have a current diagnosis of depression, and 17% of adolescents (ages 12-17) report depressive symptoms in the last two weeks (7). Autistic children and adolescents show even higher rates; a meta-analysis found that they are approximately four times more likely to experience depression than the general population (2), and in a large sample (1,272) of autistic youth, parents reported depression diagnosis for 20.2% adolescents aged 13-17 (8).

Overlapping symptoms such as flat affect, isolation, and social withdrawal, further complicate depression detection in autistic individuals (4). Some characteristics and behaviors that are considered hallmarks of autism may contribute to the increased risk of depression in them, including executive dysfunction (9–11). Executive functions (EF) encompass different domains including, but not limited to, the inhibition of prepotent responses, shifting, emotion regulation, planning, and working memory (12, 13). Inhibitory control allows us to regulate our thoughts and actions, suppress automatic responses, and ignore distracting stimuli (14). Response inhibition is essential for governing our complex thoughts and adaptive behaviors (15). In neurotypical (NT) individuals with various levels of depressive symptoms, difficulty inhibiting inappropriate behavior was found to contribute to depression due to the limitations in disengaging and in inhibiting attention from negative stimuli (16). While autistic individuals may be aware of the need to refrain from certain behaviors, they may be unable to or may have more difficulty suppressing prepotent responses (17). Autistic individuals show difficulties in both stopping processes that have already begun as well as suppressing processes before they begin (18). While repetitive thoughts can be constructive if they are positive or goal-directed, excessive negative thoughts, or depressive rumination, can lead to depression, anxiety, and poor physical health in the general population (19). An inability to disengage from negative internal stimuli can lead to increased levels of depressive rumination, which is both a symptom of and a risk factor for developing depression (20). Emotion regulation (ER) is the ability to control emotions, such as when they occur and how they are expressed (21). ER requires cognitive flexibility, set shifting, and working memory (22). In autistic children, EF deficits are linked to maladaptive coping strategies. Moreover, emotion dysregulation may be an underlying mechanism in the development of internalizing psychopathology, which is linked to the rejection from peers and victimization (12, 23). A meta-analysis by Costescu et al. (2013) quantifying the association between EF, ER, and affective problems found autistic children with fewer EF deficits had less affective problems (12).

In addition to difficulties in with EF, a characteristic often associated with autism is the difficulty in recognizing social cues or interpreting nonverbal communication cues (24–26), which negatively impacts socialization skills and outcomes (27). The behavioral theory of depression suggests that poor social skills are antecedents to depression because of limited positive reinforcements from the social environment (28). While most evidence of poor social skills and depression comes from adult populations, there are supporting findings in children as well. An investigation of nonverbal decoding abilities (i.e., interpreting nonverbal social cues) in a sample of NT children ages 7–11 found an association between deficits in this area and depression in boys (29). Using a relatively large sample of participants, one study found that among autistic children and their siblings, those who showed greater difficulty in social skills (social awareness, social cognition, social communication, and social motivation) reported more clinically significant difficulties in areas such as depression/anxiety, interpersonal relationships, and personal adjustment (30). Although autistic children and adolescents may desire social interaction and friendships, it is not uncommon for them to be rejected by their NT peers or social groups (31). Consequently, social rejection attributed to differences in social communication abilities/strategies may contribute to higher rates of psychological distress in autistic individuals. In a recent review examining risk factors for psychopathology among autistic young adults (32), the authors describe a negative feedback loop in which an autistic individual begins with an increased risk for social rejection, negative interpersonal experiences, become less motivated to seek out social interaction, and thus becomes more isolated and wary, which then reinforces the loop.

A prior study by Hollocks et al. (2014) assessed the relationship between EF and social functions with depression in autistic adolescents. In particular, they assess inference inhibition, attention switching, working memory and cognitive shifting, finding no significant associations (33). However, this is only one study, leaving room for further investigation at a broader level, including other developmental stages, groups beyond autism, and other EF. Therefore, the aim of the present study is to examine the relationship between EF and social communication and interaction with depressive symptoms in NT and autistic children and adolescents. Within EF, we assessed the domains of inhibit and self-monitor (BRI: Behavior Regulation Index), shift and emotion control (ERI: Emotion Regulation Index), as well as initiate, working memory, plan/organize, task-monitor and organization materials (CRI: Cognitive Regulation Index). We hypothesized that weaknesses in emotional, behavioral, and cognitive regulation play a role in depressive symptoms which, in turn, can lead to negative affect. We also hypothesize that weaknesses in social communication and interaction play a role in depressive symptoms due to the difficulties in socialization with peers, leading to feelings of isolation and negative affect.

Importantly, the measures employed to screen these behaviors in children are typically parent-reported rather than self-reported or direct observation. While parent-report can be advantageous, particularly when collecting large datasets, as in the present study, it may also introduce biases that influence the inferences. Relatively few studies have explored how parental internal processes shape their reports of their children’s emotional and behavioral status. Studies, thus far, have found that self-compassionate parents of autistic children and parents who have received mindfulness training view their children’s behavior in a more positive light, while parental stress contributes to the perception of the difficulties their children face (34–37). To address this potential confound, we explored the influence of parental stress in our sample to contribute to the current understanding of this growing area of research and to account for potential confounds. The findings of the current study will contribute to understanding the course of depression in NT and autistic children and adolescent populations, specifically in those who experience EF and social communication difficulties. This may guide in identifying potential risk factors for depression to monitor in autistic youth and provide insights for developing successful interventions.

## 2 Methods

### 2.1 Participants

This study included 62 autistic and 55 NT participants (see **Table 1** for demographic information). *A priori* power analyses were conducted in R using the *pwr.p.test* function to determine the sample sizes needed for the main analyses: regression models examining whether cognitive variables, executive functions and social communication and interaction, were associated with depressive symptoms. We assumed medium effect sizes (*f²* = 0.15), α = 0.05, and power = 0.80). The required total sample size was 66 participants for models including three EF predictors (*k* = 3) and 44 participants for models including one SCI predictor (*k* = 1). The final sample exceeded the minimum requirements for all analyses. In addition, participants from families who completed the Parental Distress (PD) assessment were selected as a subsample, which included 43 autistic (34 M, 9 F, mean age 10.83 ± 1.86) and 45 NT (20 M, 25 F, mean age 10.06 ± 1.68, see **Supplementary Table S.1**) participants. The study was approved by the Institutional Review Board of the University of Alabama at Birmingham. This study was part of a larger study protocol that recruited autistic and NT participants through flyers and community engagement to participate in a functional Magnetic Resonance Imaging reading comprehension intervention study. Inclusion criteria for all participants were: a full-scale intelligence quotient (FSIQ) and Verbal Comprehension Index (VCI) of 70 or higher, 7–13 years of age, no history of seizures or epilepsy, and the ability to complete an MRI scan. FSIQ and VCI scores were obtained using the Weschler Abbreviated Scale of Intelligence, 2^nd^ Edition (WASI-II) (38). All participants also met the following medication guidelines: no antipsychotics for at least one month, no anti-convulsant for at least one week, no stimulants for 24 hours prior to testing, and a stable dose of SSRIs for at least two weeks. The presence of comorbid conditions was reported by some participants, including conditions such as ADHD and anxiety (detailed information on comorbidity profiles for each group is provided in **Supplementary Table 2**). Participants were excluded if they did not meet any of the above inclusion criteria and medication guidelines, or if they were unable to complete the testing and MRI session. Socioeconomic status data were not collected from the participants in this study.

**Table 1:** Demographics for full sample.

Descriptive statistics	ASD (n=62)	NT (n=55)	p-value
Gender			0.0003
Male	50	26	
Female	12	29	
Age	10.71 ± 1.93	9.92 ± 1.74	0.13
FSIQ	87.43 ± 11.95	107.98 ± 16.24	0.0001
SRS-2	75.13 ± 10.71	44.53 ± 09.05	0.0001
ADOS-2 (n = 8)	9 ± 1.07	NA	NA
BOSA (n = 49)	9.39 ± 2.78	NA	NA
ADI-R (n = 53)			
Reciprocal Social Interaction	20.02 ± 6.01	NA	NA
Communication	16.28 ± 4.58	NA	NA
Repetitive Behavior	4.91 ± 2.31	NA	NA
Abnormality of Development	3.57 ± 1.39	NA	NA
SCQ	17.37 ± 6.80	2.90 ± 3.67	0.0001

the p-value for gender was calculated using chi squared and for age a t test was used. The self-identity of the subjects was mainly white or Caucasian (ASD = 28, NT = 35), followed by black or African American (ASD = 17, NT = 12), Asian (ASD = 14, NT = 5), more than one (ASD = 2, NT = 3), and did not specify (ASD = 1). ASD, Autism Spectrum Disorder; NT; Neurotypical; FSIQ, Full-Scale Intelligence Quotient, SRS-2, Social Responsiveness Scale, 2nd Edition, ADOS-2, Autism Diagnostic Observation Schedule; BOSA, Brief Observation of Symptoms of Autism, ADI-R, Autism Diagnostic Interview-Revised; SCQ, Social Communication Questionnaire. Participants without explicitly stated sample sizes contributed full data for the corresponding variables, and all available data were included in the reported results.

All autistic participants had a previous clinical diagnosis of ASD, which was corroborated by a combination of diagnostic measures obtained in this study and expert opinion from a licensed clinical psychologist. These diagnostic measures were administered *only* to the autistic participants and were the Autism Diagnostic Interview Revised (ADI-R) (39), the Autism Diagnostic Observation Schedule, 2^nd^ Edition (ADOS-2) (40, 41), and the Brief Observation of Symptoms of Autism (BOSA) (42). While we planned administering ADOS-2 on all our participants, we had to switch to a different instrument after the first 8 autistic participants of this study. For the rest of the participants, we used BOSA in order to accommodate health and safety guidelines during the COVID-19 pandemic. We continued to use the BOSA even after COVID-19-related restrictions were removed, due to the high number of participants who had already been given the BOSA instead of the ADOS-2. Acknowledging the differences in administration and criteria for these measures, we included an opportunity for expert opinion to override the diagnostic assessment results to determine inclusion in the ASD group. Participants were first assessed using the ADOS-2/BOSA and the ADI-R; if a participant met the diagnostic cutoff for one of these assessments, but not the other, they were still included in the ASD group. There was no specific “level” of diagnosis required, and ASD symptom severity varied across participants (9 ± 1.07 on the ADOS-2 total comparison score; 9.39 ± 2.78 on the BOSA total score); however, those with more extreme support needs typically did not end up meeting either the FSIQ/VCI cut-off score, or had issues related to the MRI scan. Thus, most autistic participants had either a Level 1 or Level 2 diagnosis, as described by the DSM-V. All NT participants were medically healthy and scored below ASD symptom cutoff score on the Social Communication Questionnaire (SCQ) (43); the SCQ was also administered to autistic participants, but was not considered an inclusion criterion. Participants’ legal guardians signed a written informed consent, and participants gave written and verbal assent.

The above-mentioned inclusion criteria are part of a larger study protocol from which the present sample was drawn. To prevent any bias resulting from the impact of extreme values, an outlier detection analysis was conducted on the data using R statistical software (44), specifically the ‘*rstatix*’ library (45). Outliers are defined as values that fall beyond the range of the third quartile + 1.5 of the interquartile range, or below the first quartile + 1.5 of the interquartile range. Following the identification of outliers in the data, five subjects were excluded from further consideration in this study. Of these subjects, four were from the autistic group and one was from the NT group.

### 2.2 Behavioral measures

Participants’ caregivers completed questionnaires on their child’s behaviors and emotions which evaluated symptoms associated with depression, executive functioning, and social skills. The Behavior Assessment System for Children, Third Edition (BASC-3) (46, 47) was administered to parents, which assesses the behaviors and emotions of participants. The BASC-3 consists of 175 questions for the Child form (administered to participants ages 7-11), and 173 questions on the Adolescent form (administered to participants aged 12-13). The depression scale within the BASC-3 was used to assess depressive symptoms with a higher t-score meaning a clinically significant level of maladaptive behavior which may meet diagnostic criteria. The Behavior Rating Inventory of Executive Function, Second Edition (BRIEF-2) (48) was used to assess domains of EF, such as behavioral regulation, emotional regulation, and cognitive regulation. The BRIEF-2 consists of 63 items contributing to nine subscales which can be grouped into three indices: behavior, emotion, and cognitive regulation, which were used in the statistical analysis. The BRI describes an individual’s ability to modulate and monitor their own behavior, which includes inhibit and self-monitor scales. The ERI describes an individual’s ability to manage emotional responses and adapt to changes and includes shift and emotion control scales. The CRI describes an individual’s ability to control cognitive processes and utilize problem-solving skills, including working memory, plan/organize, task-monitor and organization of materials scales. BRIEF-2 raw scores are converted to t-scores using age and sex norms, with higher scores on all measures indicating more severe deficits in their respective realms (48). To measure social communication and interaction, the Social Responsiveness Scale, Second Edition (SRS-2) (49) was used. This parent-report questionnaire identifies the presence and severity of social impairments in autism. The SRS-2 consists of five behavioral subscales: Social Awareness, Social Cognition, Social Communication, Social Motivation, and Restricted/Repetitive Behaviors/Interests. The Social communication and interaction index (SCI), which includes all subscales of the SRS except Restricted/Repetitive Behaviors/Interests was used in the present statistical analysis. SRS-SCI-2 raw scores are converted to t-scores that account for sex norms, with higher t-scores indicating greater difficulty in that area. The Parenting Stress Index, 4^th^ Edition Short Form (PSI-4) (50) was administered to caregivers to measure domains of parental stress: Parental Distress, Parent-Child Dysfunctional Interaction, and Difficult Child. For this study, only the Parental Distress domain was used in analyses to isolate the parent’s emotional state from their child’s behavior and measure the levels of stress experienced by parents. PSI-4 raw scores are converted to t-scores for use in analysis.

### 2.3 Statistical analysis

The following steps were performed using R (44). Descriptive statistics were calculated for each of the measures, and the resulting means and standard deviations are presented in the following sections. Comparisons between the groups were conducted using t-test analyses. Subsequently, to ascertain whether EF and SCI were associated with symptoms of depression; linear models were constructed for each of these variables. The initial model incorporated EF, encompassing the BRI, ERI, and CRI of the BRIEF-2. The second model incorporated the SCI subscale of the SRS-2. FSIQ was included as a covariate in both models. To test the interaction of these variables in different populations, the models were tested first including only the NT group, a second model included the autistic group, and the third model included both groups.

The current study utilized indirect measures (i.e., parent report questionnaires) to assess the cognitive processes of interest, including depressive symptoms, EF, and social communication and interaction. Therefore, we investigated whether such measures may be biased by the perception of the individual responding to the inventory. To assess whether parental distress influenced the results of the social and EF measures, we ran the multilinear models previously described, with Parental Distress added as a moderator variable. For this analysis, we used a subsample that completed the parental distress measure (see **Table A.1**). The significance level was set at p < 0.05, and the Bonferroni correction was applied for multiple comparisons (between models = 0.05/12 = 0.004, within models = 0.05/3 = 0.016 for EF and 0.05 for SCI). Furthermore, the regression linear assumptions were assessed for the models, encompassing the normality of residuals, homoscedasticity, independence of errors, multicollinearity, and outliers. These evaluations were conducted in R, with the performance package (51). In the event that a model exhibited failure in one of these assumptions, a Generalized Additive Model (gam; cubic spline basis for SCI: gam (depression ~ s(SCI) + FSIQ + age)), a nonparametric method for regression models, using the gam function from the mgcv library (52).

## 3 Results

### 3.1 Group differences

**Figure 1:**
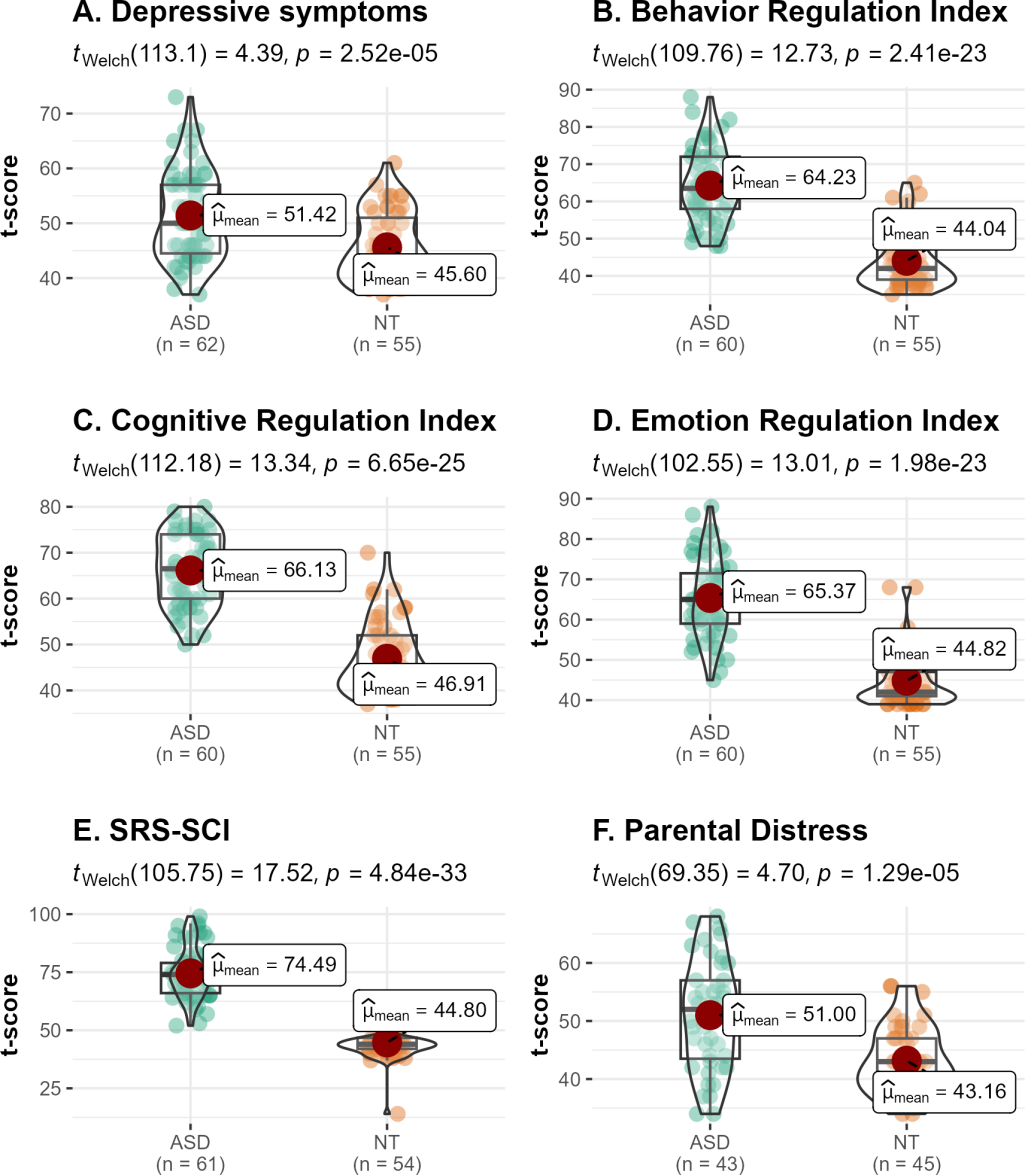
Group differences in behavioral and cognitive measures. Autistic children showed higher **(A)** Depressive symptoms; more difficulties in the **(B)** Behavior Regulation Index, **(C)** Cognitive Regulation Index, **(D)** Emotion Regulation Index, and greater alterations in **(E)** Social Communication & Interaction. In addition, parents of autistic children showed greater **(F)** Parental Distress. Data points for autistic participants are shown in green and for neurotypical participants in orange. All measures are presented as T-scores.

The autistic participants showed significantly elevated depressive symptoms (51.42 ± 8.04) in comparison to NT participants (45.60 ± 6.25). Seven autistic participants and one NT participant scored in the “at-risk” range for depression (T = 60–69), and one autistic participant scored in the clinically significant range (T = 73; T ≥ 70 indicates clinical significance). Autistic participants also showed greater challenges in EF in the three measures. In particular, the BRI (autistic: 64.23 ± 9.58; NT: 44.04 ± 7.37), the ERI (autistic: 65.36 ± 10.10; NT: 44.81 ± 6.61), and CRI (autistic: 66.13 ± 7.72; NT: 46.91 ± 7.71) scores were significantly higher in autistic, relative to NT, participants. The SCI also exhibited significantly elevated scores among the autistic group (74.49 ± 10.77) in comparison to the NT (44.79 ± 7.25). Finally, parents of autistic children demonstrated higher levels of distress (51.00 ± 9.37) in comparison to parents of NT children (43.15 ± 5.78; see **Figure 1**).

### 3.2 Executive functioning

The linear regression models indicated that EF was a statistically significant predictor of depressive symptoms in the models that included only NT group (*F*(4,50) = 6.08, *p* = 0.0004, *adjusted-R^2^* = 0.27), only the autistic group (*F*(4,55) = 9.81, *p* < 0.0001, *adjusted-R^2^* = 0.37), and the model incorporating both groups (ASD + NT) (*F*(4,110) = 22.73, *p* < 0.0001, *adjusted-R^2^* = 0.43). The model explaining the greatest proportion of variance was the one including both samples (45.25%), followed by the ASD-only model (41.65%) and the NT-only model (32.75%). Across the three models, the only significant predictor was the ERI: both groups (*β* = 0.45, *t* = 5.64, *p* < 0.0001), ASD (*β* = 0.48, *t* = 4.59, *p* < 0.0001), and NT (*β* = 0.53, *t* = 3.39, *p* = 0.001), accounting for a substantial proportion of the variance in each model (24.26% in the combined model, 27.78% in the ASD model and 21.32% in the NT model). In contrast, the BRI and CRI were not significant predictors in any of the three models (see **Figure 1A**).

### 3.3 Social communication and interaction

**Figure 2:**
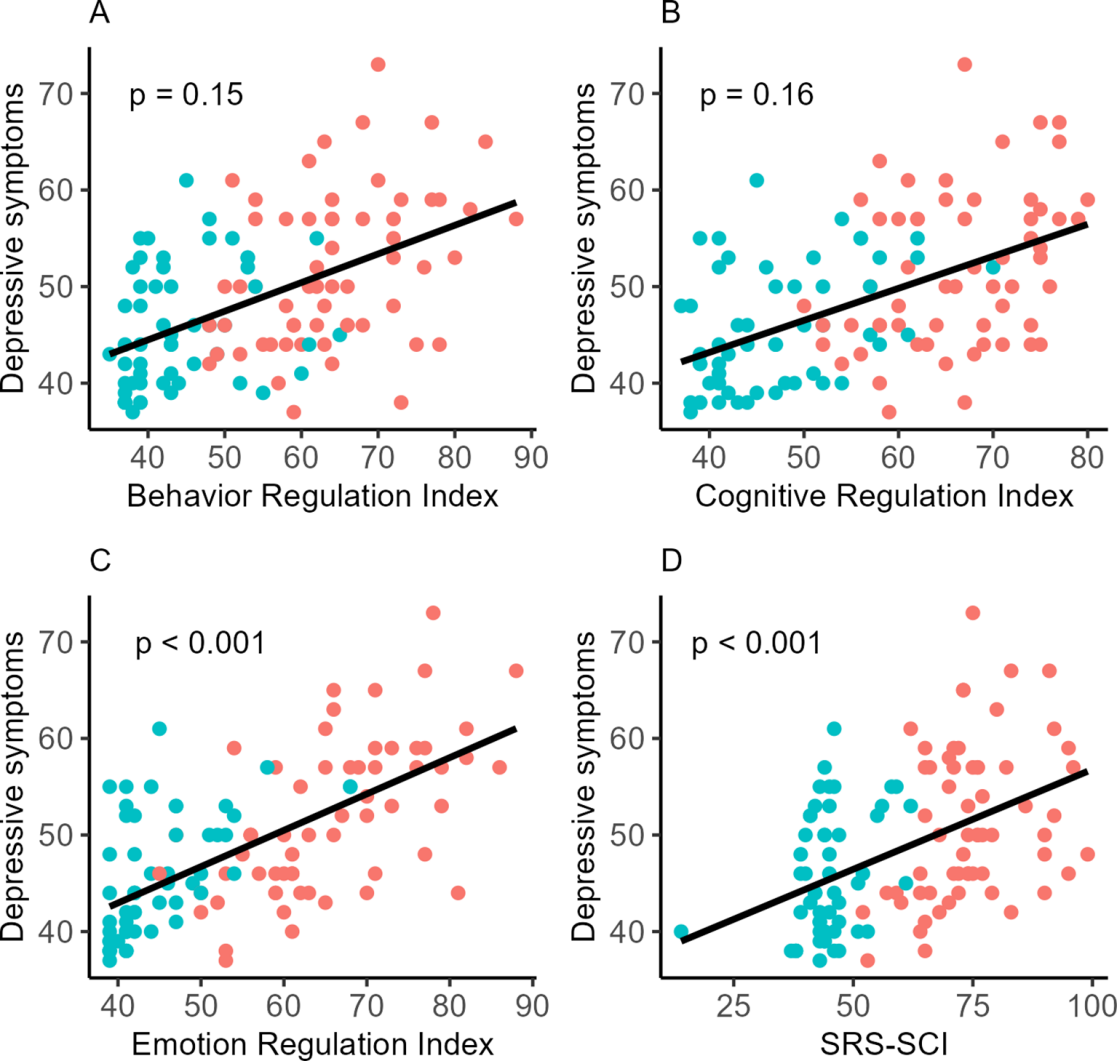
Relationship between depressive symptoms and the predictors. **(A)** Behavior Regulation Index, **(B)** Cognitive Regulation Index, **(C)** Emotion Regulation Index, and **(D)** Social Communication & Interaction (SRS-SCI*).* Data points for autistic participants are shown in red and for neurotypical participants in blue. All measures are presented as T-scores. Depressive symptoms were not significantly predicted by Behavior Regulation Index and Cognitive Regulation Index.

The linear regression involving NT participants indicated that SCI was a significant model for predicting depressive symptoms (*F*(3,50) = 3.42, *p* = 0.02, *adjusted-R^2^* = 0.12), but did not survive Bonferroni correction. Similarly, the SCI was not identified as a significant predictor of depressive symptoms when only the ASD group was included in the analysis (*F*(3,57) = 2.26, *p* = 0.09, *adjusted-R^2^* = 0.06). However, the results indicated that the SCI was a significant predictor when both autistic and NT groups were included in the model (*F*(3,111) = 10.57, *p* < 0.0001, *adjusted-R^2^* = 0.20; see **Figure 2C**), explaining 21.78% of the variance in depressive symptoms when both groups were included in the model. The only significant predictor was the SRS-SCI (*β* = 0.21, *t* = 4.84, *p* < 0.0001), which explained 19.31% of the variance in depressive symptoms in the autistic and NT samples (see **Figure 2D**, **Supplementary Figure S1B**). The model that included both groups revealed non-normality in the residuals, therefore we performed a Generalized Additive Model, which corroborated the significance of the SCI to explain symptoms related to symptoms when using both samples.

### 3.4 Effect of parental distress as moderator

Given that the tests employed to evaluate EF and SCI are completed by the primary caregiver and considering that parental distress may influence the results of the regression models, we re-ran the linear models including parental distress as moderator variable. When parental distress was incorporated, the NT model (*F*(8,36) = 3.09, *p* = 0.009, *adjusted-R^2^* = 0.27) and the ASD group model (*F*(8,34) = 3.97, *p* = 0.002, *adjusted-R^2^* = 0.36) were statistically significant; and remained significant after Bonferroni correction. Moreover, no individual predictors reached significance in either model. The model with both groups was significant (*F*(8,79) = 9.46, *p* < 0.001, *adjusted-R^2^* = 0.43), but no individual predictors were statistically significant (see **Supplementary Figure S1A**).

**Figure 3:**
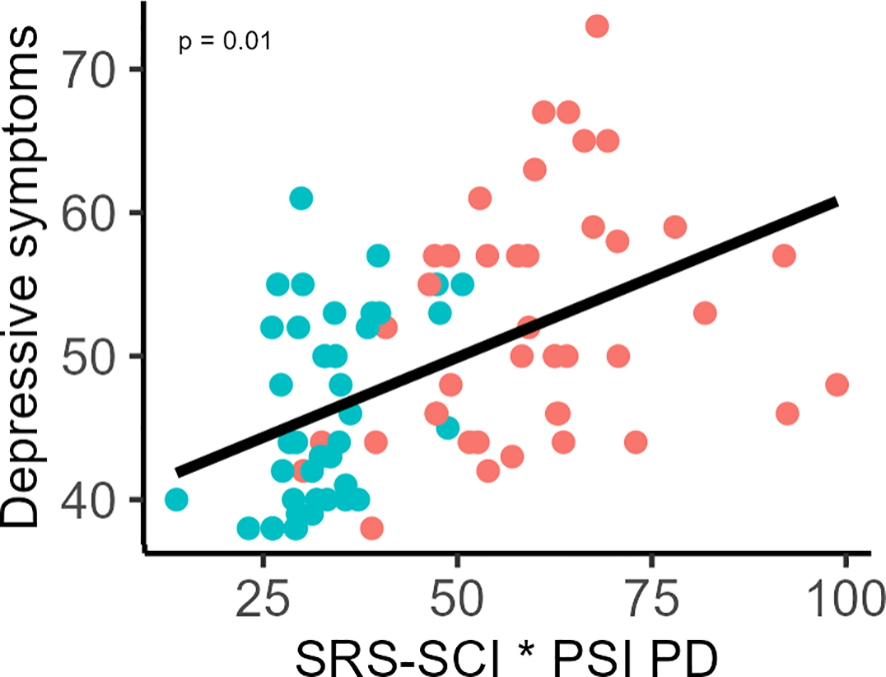
Moderating effect of Parental Distress (PSI-PD) on the association between depressive symptoms and Social Communication & Interaction (SRS-SCI) scores. Higher parental distress strengthens the relationship between depressive symptoms and SRS-SCI difficulties. Data points for autistic participants are shown in red and for neurotypical participants in blue. All measures are presented as T-scores.

For the SCI, the results revealed that when PD was added as a moderator the models remained non-significant for NT (*F*(5,38) = 2.36, *p* = 0.06, *adjusted-R^2^* = 0.13) and autistic samples (*F*(5,36) = 2.65, *p* = 0.04, *adjusted-R^2^* = 0.17). The model including both groups remained significant (*F*(5,80) = 7.93, *p* < 0.0001, *adjusted-R^2^* = 0.29), explaining 33. of the variance in depressive symptoms. Within this model, SCI continued to emerge as a significant individual predictor (*β* = 0.76, *t* = 2.92, *p* = 0.004). Furthermore, PD was a significant predictor (*β* = 1.18, *t* = 3.27, *p* = 0.001), and its interaction with SCI was also significant (*β* = -0.01, *t* = -2.53, *p* = 0.01, see **Figure 3**). The SCI explained 11.60%, the parental distress 14.57%, and the interaction between SCI with parental distress 5.30% of the variance in depressive symptoms when including both samples.

## 4 Discussion

The primary goal of this study was to gain further insights into the cognitive processes and potential risk factors associated with depressive symptoms in autistic youth. This work offers a novel contribution by being the first, in our knowledge, to jointly examine caregiver-reported EF and SCI as predictors of depressive symptoms in autistic and NT children, while also evaluating whether parental distress biases these associations. Because caregiver-report inventories are among commonly used tools in both clinical practice and scientific research for identifying emotional and behavioral difficulties, understanding the validity and potential sources of bias in these measures is highly relevant for accurate assessment in autism. Given the high prevalence of depression in autistic individuals, understanding the relationship between these variables is critical. It is even more important, considering that depression may go undiagnosed in autistic individuals due to the difficulty in distinguishing between autistic and depressive symptoms. A significant increase in depressive symptoms among autistic, compared to NT, children was found in this study, which is consistent with previous findings; autistic individuals, including children, are 4 times more likely to develop depression when compared to the typical population (1–4). Furthermore, the results showed EF, in particular ERI and SCI were significantly associated with depressive symptoms across all participants. A moderation analysis was conducted to assess whether the results could be biased by the perception or emotional status of the parents responding to the questionnaires. More specifically, we ran a moderation analysis with parental distress scores as the moderator to see if stress as a parent impacts the way they respond to the questionnaire. It should be noted that some of the items within the Parental Distress scale are similar to symptoms of depression; therefore, responses to these items may be influenced by depression unrelated to the child or parental experience.

This study found greater difficulties in EF abilities among autistic, compared to NT, children across the behavior, emotion, and cognitive indices. These findings are consistent with previous literature reporting EF dysfunction in autism (9, 17, 18). In the present study, emotion regulation was significantly associated with depressive symptoms; higher scores on the ERI (indicating more severe deficits) were found to be significant predictors of depression in across the NT only group, autistic, and autistic and NT groups together. This is the first study, to our knowledge, that assessed the association between emotion regulation and depressive symptoms in autistic populations. These results align with previous longitudinal work that has shown deficits in inhibiting negative information, such as rumination, increase the likelihood of major depressive episodes by contributing to maladaptive emotion regulation in adolescence (20). Importantly, the relations between ER and depressive symptoms were not influenced by parental distress, suggesting that the association reflects direct cognitive-emotional mechanisms rather than reporting biases. This strengthens the interpretation that diminished executive control over emotional processes represents a critical pathway linking EF dysfunction to internalizing psychopathology in both autistic and NT populations.

Against our hypothesis, neither BRI nor CRI predicted depressive symptoms in either group. The BRI primarily captures inhibitory control and self-monitoring, while the CRI reflects higher-order executive processes such as working memory, planning/organization, task monitoring, and organization of materials. Similarly, studies with non-clinical adults have reported associations between inhibitory control and depressive symptoms (16). In contrast, and consistent with our findings, Hollocks et al. (2014) reported no significant associations between inhibitory control, working memory, and depressive symptoms in autistic adolescents. Several factors may account for the lack of associations observed here. One possibility is that participants in both groups reported relatively low levels of depressive symptoms, which may have reduced the variability necessary to detect significant relationships. Another explanation is that the relationship between EF and depression emerges more clearly in the context of clinically significant depression, where impairments in working memory, inhibitory control, and related processes are more pronounced. Future research including participants with a wider range of symptom severity as well as more fine-grained assessments of EF subdomains, will be critical to clarifying the extent to which regulatory processes contribute to depression risk in autistic and NT populations.

The results of this study also align with previous reports of autistic children exhibiting greater difficulties in SCI, such as recognizing and understanding nonverbal cues in social settings, when compared to NT children (24–26). Furthermore, the linear model, which included both groups, indicated that symptoms associated with depression could be predicted by weaknesses in SCI skills. Previous evidence has demonstrated a correlation between higher social difficulties and the presence of depression, anxiety, social stress, a greater sense of inadequacy, and difficulties in interpersonal relationships in autistic young adults (30). Individuals who are at risk of heightened deficits in social skills also have a heightened risk of social rejection, which can result in greater isolation thus creating a negative feedback loop (32). There are reports that social difficulties in autistic students are positively related to bullying experiences, and such experiences of bullying and victimization relate to the later development of psychopathology (53). This may subsequently result in an increase in depressive symptoms. Our findings are consistent with this framework, suggesting that individuals with poorer social skills are more likely to experience greater symptoms of depression. Importantly, these associations remained significant in the parental distress moderator, underscoring the robustness of the SCI–depression association. Interestingly, this relationship was evident when both groups were analyzed together but not when examined separately, suggesting that variability in SCI across broader samples may provide greater statistical power to detect such associations, whereas within-group models may be constrained by more restricted ranges of social functioning. This pattern highlights the importance of considering both within- and across-group variability when modeling relationships between social difficulties and mental health outcomes. It’s noteworthy that the parental distress moderated the relationship between depressive symptoms and SCI. Specifically, higher parental distress amplified the association between depressive symptoms and SRS-SCI in both groups. This influence could be due to multiple reasons, including that parents who are distressed may perceive and report their child’s symptoms differently, amplifying the observed relationship between SCI and depression. Another possibility is that children’s risk for depressive symptoms may reflect not only their own social challenges but also the broader emotional climate of the family. Future work could explore this.

A limitation of this study related to the diagnostic tool used to corroborate autism diagnoses. Due to COVID-19 related constraints, diagnostic procedures varied across participants, with some evaluated using ADI-R, others with the BOSA, and a smaller subset with ADOS. Another limitation is the sample size, which precludes a subgroup analysis by age group, including children and adolescents. It has been documented that depressive symptoms typically increase during adolescence, a phenomenon that is particularly pronounced in autistic individuals (2). Therefore, further studies could investigate the impact of these cognitive processes on depression-associated symptoms in autistic and non-autistic children and adolescents. Additionally, the present sample included autistic children without intellectual disability and with adequate verbal skills, due to the inclusion criteria of the larger study protocol. Thus, the present results are only generalizable to autistic children who are cognitively able and who communicate verbally. Future studies could further examine depressive symptomology in nonverbal or individuals with verbal deficits and investigate the accurate screening of depression within that population; the same avenues should also be explored in autistic children with an intellectual disability. Another limitation to the current study includes the usage of indirect measures to assess EF, social communication and interaction skills, and depressive symptoms. As part of the larger study protocol, parents of the participants filled out questionnaires regarding their child’s behaviors and emotions. All key constructs of the current study (depressive symptoms, executive functions, and social communication) were assessed solely by parent-report questionnaires (BASC-3, BRIEF-2, SRS-2). The use of questionnaires can increase the likelihood of the common method bias. Parents may be motivated to “fake good,” or even be unmotivated to read questions carefully and choose the first response that catches their eye; this can bias report accuracy. Future studies could use direct and indirect measures in child and adolescent populations for further investigating the cognitive mechanisms underlying depression and directly assess if there are differences between both approaches.

In conclusion, the present study highlights the dual contributions of emotional regulation and SCI to depression in both autistic and NT populations. By integrating EF, SCI and parental distress into a single model, our findings offer novel evidence that emotion regulation deficits, particularly those captured by the ERI, including shifting and emotional control, are robust predictors of depressive symptoms and remain significant even after accounting for parental reporting biases. SCI difficulties were also associated with depressive symptoms; although this association remained significant in the moderation analysis, the strength of the relationship was influenced by parental distress, with higher parental distress amplifying the SCI–depression relationship. Given that parent-report inventories are widely used in both clinical and research settings, these findings reinforce their relevance while underscoring the importance of careful interpretation. These findings underscore the importance of addressing emotion regulation and social communication in identifying risk factors of depression and developing intervention strategies for depression.

## Data Availability

The raw data supporting the conclusions of this article will be made available by the authors, without undue reservation.

## Supplementary material

SUPPLEMENTARY FIGURE 1 Regression model results examining the association between depression symptoms and cognitive resources. Panel A displays models testing the relationship between depression and executive functions, and Panel B shows models testing the relationship between depression and Social Communication and Interaction (SCI). For each cognitive domain, six regression analyses were conducted: three using separate samples (NT only, ASD only, and combined ASD+NT), and three parallel analyses including parental distress (PSI–Parental Distress) as a moderating variable in each sample. Significant models are indicated with asterisks (p = 0.05*, 0.01**, 0.001***). NT = neurotypical; ASD = autism spectrum disorder; PSI–PD = Parenting Stress Index, Parental Distress; BRIEF = Behavior Rating Inventory of Executive Function; BRI = Behavior Regulation Index; CRI = Cognitive Regulation Index; ERI = Emotion Regulation Index; SRS–SCI = Social Responsiveness Scale, Social Communication & Interaction; FSIQ = full-scale intelligence quotient.
