# Supplementary figures and images for: Symptoms of depression in autistic children and adolescents
Source: Front Psychiatry. 2025 Dec 15;16:1697147. doi: 10.3389/fpsyt.2025.1697147 (PMC12745227; doi:10.3389/fpsyt.2025.1697147)

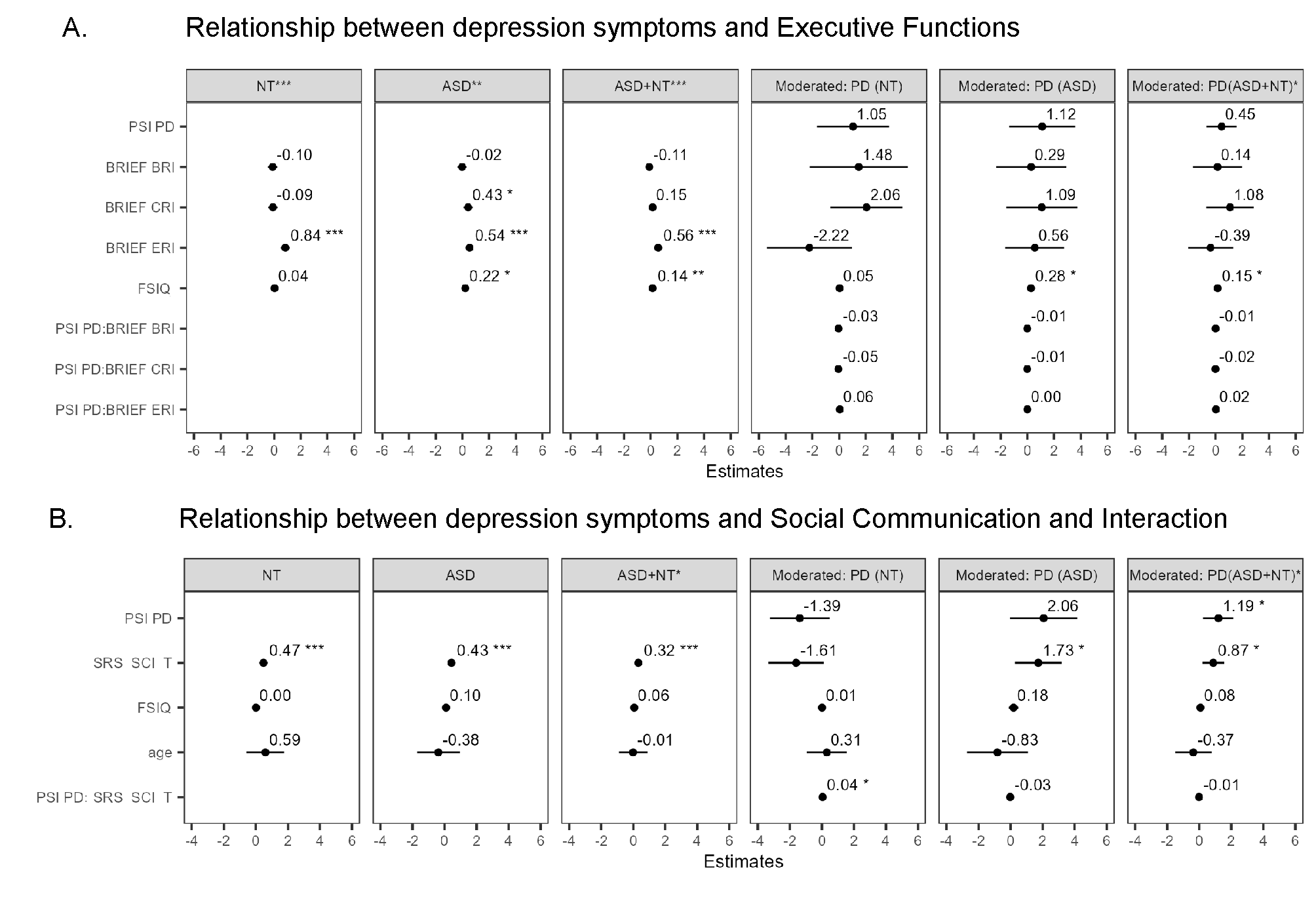

Supplement: SUPPLEMENTARY FIGURE 1 — Regression model results examining the association between depression symptoms and cognitive resources. Panel A displays models testing the relationship between depression and executive functions, and Panel B shows models testing the relationship between depression and Social Communication and Interaction (SCI). For each cognitive domain, six regression analyses were conducted: three using separate samples (NT only, ASD only, and combined ASD+NT), and three parallel analyses including parental distress (PSI–Parental Distress) as a moderating variable in each sample. Significant models are indicated with asterisks (p = 0.05*, 0.01**, 0.001***). NT = neurotypical; ASD = autism spectrum disorder; PSI–PD = Parenting Stress Index, Parental Distress; BRIEF = Behavior Rating Inventory of Executive Function; BRI = Behavior Regulation Index; CRI = Cognitive Regulation Index; ERI = Emotion Regulation Index; SRS–SCI = Social Responsiveness Scale, Social Communication & Interaction; FSIQ = full-scale intelligence quotient. [file Image1.tif]
