# Supplementary material for: Symptoms of depression in autistic children and adolescents
Source: Front Psychiatry. 2025 Dec 15;16:1697147. doi: 10.3389/fpsyt.2025.1697147 (PMC12745227; doi:10.3389/fpsyt.2025.1697147)
Supplement: Supplementary file 2 [file Table1.docx]

**Table S.1.** Demographics for parental distress moderator analysis

| **Descriptive Statistics** | **ASD**  **(n=29)** | **NT**  **(n=19)** | **p-value** |
| --- | --- | --- | --- |
| ***Gender*** |  |  | 0.01 |
| *Male* | 24 | 8 |  |
| Female | 5 | 11 |  |
| ***Age*** | 10.69 ± 1.85 | 9.88 ± 1.72 | 1.0 |
| ***FSIQ*** | 88.48 | 111.60 | 0.0001 |
| ***SRS*** | 75.98 | 46.84 | 0.0001 |

Note: the p-value for gender was calculated using chi squared and for age a t test was used. The self-identity of the subjects was mainly white or Caucasian (ASD = 15, NT = 11), followed by Asian (ASD = 9, NT = 4), black or African American (ASD = 4, NT = 3) and more than one (ASD = 1, NT = 1). ASD = Autism Spectrum Disorder, NT = Neurotypical.
