# Supplementary material for: Symptoms of depression in autistic children and adolescents
Source: Front Psychiatry. 2025 Dec 15;16:1697147. doi: 10.3389/fpsyt.2025.1697147 (PMC12745227; doi:10.3389/fpsyt.2025.1697147)
Supplement: Supplementary file 3 [file Table2.docx]

**Table S.2.** Comorbidities reported by group

| **Comorbidities** | **ASD**  **(n = 62)** | **NT**  **(n = 55)** |
| --- | --- | --- |
| No comorbidities | 32 | 52 |
| ADD, anxiety | 1 | 0 |
| ADHD | 12 | 1 |
| ADHD, anxiety | 5 | 0 |
| ADHD, hypotonia | 1 | 0 |
| ADHD, language disorder | 2 | 0 |
| ADHD, learning disability | 1 | 0 |
| ADHD, learning disability, anxiety | 1 | 0 |
| Anxiety | 2 | 0 |
| Asthma | 0 | 1 |
| Auditory processing disorder | 0 | 1 |
| Chron's disease | 1 | 0 |
| ADHD, anxiety and dyslexia | 1 | 0 |
| Language Impairment | 1 | 0 |
| Sensory Processing Disorder | 1 | 0 |
| SLD in Basic Reading | 1 | 0 |

ADD = Attention Deficit Disorder, ADHD = Attention Deficit Hyperactivity Disorder, SLD = Specific Learning Disorder.
